# Organ-specific transcriptome analysis reveals differential gene expression in different castes under natural conditions in *Apis cerana*

**DOI:** 10.1038/s41598-021-90635-3

**Published:** 2021-05-28

**Authors:** Igojo Kang, Woojin Kim, Jae Yun Lim, Yun Lee, Chanseok Shin

**Affiliations:** 1 Department of Agricultural Biotechnology, Seoul National University, Seoul, 08826 Republic of Korea; 2 Department of Agricultural Biology, Jeonbuk National University, Jeonju, 54896 Republic of Korea; 3 Department of Applied Biology and Chemistry, Seoul National University, Seoul, 08826 Republic of Korea; 4 Research Institute of Agriculture and Life Sciences, Seoul National University, Seoul, 08826 Republic of Korea; 5 Plant Genomics and Breeding Institute, Seoul National University, Seoul, 08826 Republic of Korea

**Keywords:** Computational biology and bioinformatics, Molecular biology

## Abstract

Honeybees are one of the most environmentally important insects, as their pollination of various plant species contributes to the balance among different ecosystems. It has been studied extensively for their unique attribute of forming a caste society. Unlike other insects, honeybees communicate socially by secreting pheromones or by exhibiting specific patterns of motion. In the honeybee industry, the Asian honeybees (*Apis cerana*) and the Western honeybees (*Apis mellifera*) are dominant species. However, molecular research on the transcriptomes of *A. cerana* has not been studied as extensively as those of *A. mellifera.* Therefore, in this study, caste-specific transcriptional differences were analyzed, which provides a comprehensive analysis of *A. cerana*. In our dataset, we analyzed gene expression profiles using organs from worker, drone, and queen bees. This gene-expression profile helped us obtain more detailed information related to organ-specific genes, immune response, detoxification mechanisms, venom-specific genes, and ovary development. From our result, we found 4096 transcripts representing different gene-expression pattern in each organ. Our results suggest that caste-specific transcripts of each organ were expressed differently even under natural conditions. These transcriptome-wide analyses provide new insights into *A. cerana* and that promote honeybee research and conservation.

## Introduction

Considering the vital role of honeybees in supporting ecosystems and thus sustaining lifeforms, including human life, honeybees are irreplaceable by other insects^1,2^, because they are the largest group crop pollinators^3,4^ and provide honey^5,6^, beeswax, propolis^7^, royal jelly^8^, and many other benefits^9–11^. A honeybee colony is composed of a single queen and thousands of workers and a couple of hundred drones, which provides a classic model for understanding the sociality^12–16^, communication^17,18^, and sex-determination^19–21^ of insects. Genetically, females, queen and worker bees, develop from fertilized eggs, whereas drones develop from unfertilized eggs^22^. Previous studies of honeybees described a simple genetic system of haplodiploid development and showed that different diets and cell sizes determine the physiological characteristics, such as body size, behavior, physiology, and lifespan^23^, of the three castes of worker, drone, and queen bee. The relationship between honeybees behavior and the gene expressions^24^ of odorant receptors^25–27^ and hormones^28,29^ in the brains of workers and queen bees were investigated in numerous studies that investigated the social aspect of bee behavior. While developing into three castes, royal jelly was revealed to trigger larvae to develop into a queen and control the gene expression of the juvenile hormone, which plays a key role in sex-determination^30,31^.

Honeybees live in a densely populated environment, have close connections, and share food with nest-mates^32^. This causes pathogens to spread easily and very quickly within the colony. Therefore, honeybees are highly susceptible to pathogens. Researchers have focused to understand how honeybees resist disease and protect their health. Honeybees have evolved two strategies for protecting their colonies from pathogens; (1) an innate immune system^33,34^ and (2) social immunity^35,36^. First, honeybees protect their colony by protecting themselves, which is achieved with an innate immune system. As one example, workers are major pollinators that forage on wildflowers and crops. While foraging, honeybees are exposed to harmful agricultural pesticides and pathogen, such as viral and bacterial infections^37^, which may be transmitted to the rest of the bee colony. Studies on the innate immune system of bees have elucidated the stronger innate immune response of younger forger bees compared to older forager bees^38^. Second, the social immunity of bees has been studied as a function of health maintenance. Worker bees detect smells and remove diseased or infected brood/adults, get rid of dead adults, foreign objects, and pathogens from their hive, and clean the surface of their body^35,36^.

Honeybees are considered interesting social insect models and have been extensively studied on the subjects such as insect-communication system, flight behavior, and developmental biology. Despite these diverse studies on honeybees, there is still insignificant understanding of the sex determination of haplodiploid system at the molecular level. There are two genetically same types of female honeybees: worker bees and the queen bee. Worker bees can lay eggs usually in the absence of a queen. Unlike the queen that can mate and store sperms in spermatheca to lay fertilized eggs^39^, worker bees cannot mate. Therefore, only the queens are able to lay fertilized eggs whereas workers’ eggs remain unfertilized^40,41^. Egg-viability studies have shown that sex determination is associated with a difference in cell size and dietary habits. Most research into sex determination has focused on phenotypes during honeybee development and is limited to studying the early stages or the organ levels^22,42^. Therefore, it would be highly informative to investigate the dynamics of expressions from the two kinds of honeybee ovaries, i.e., queen ovary and worker ovary, which could provide insights into the haplodiploid development of honeybees.

In this study, organ-specific RNA-seq was performed to analyze transcriptome data of several organs (one from the queen, five from a worker, and five from a drone) of the Asian honeybees. The gene expressions of worker-drone organs, worker-queen organs, and drone-queen organs were compared and a dataset of differentially expressed genes was validated using quantitative reverse transcription polymerase chain reaction (qRT-PCR). Specifically, our analyses focused on the expressions of genes related to the mechanisms of odorants, vision, hormones, growth factors, the immune-response, detoxification, venom, and sex determination.

## Results

### Transcriptome profiling of organs from worker, drone, and queen of *Apis cerana*

**Figure 1:**
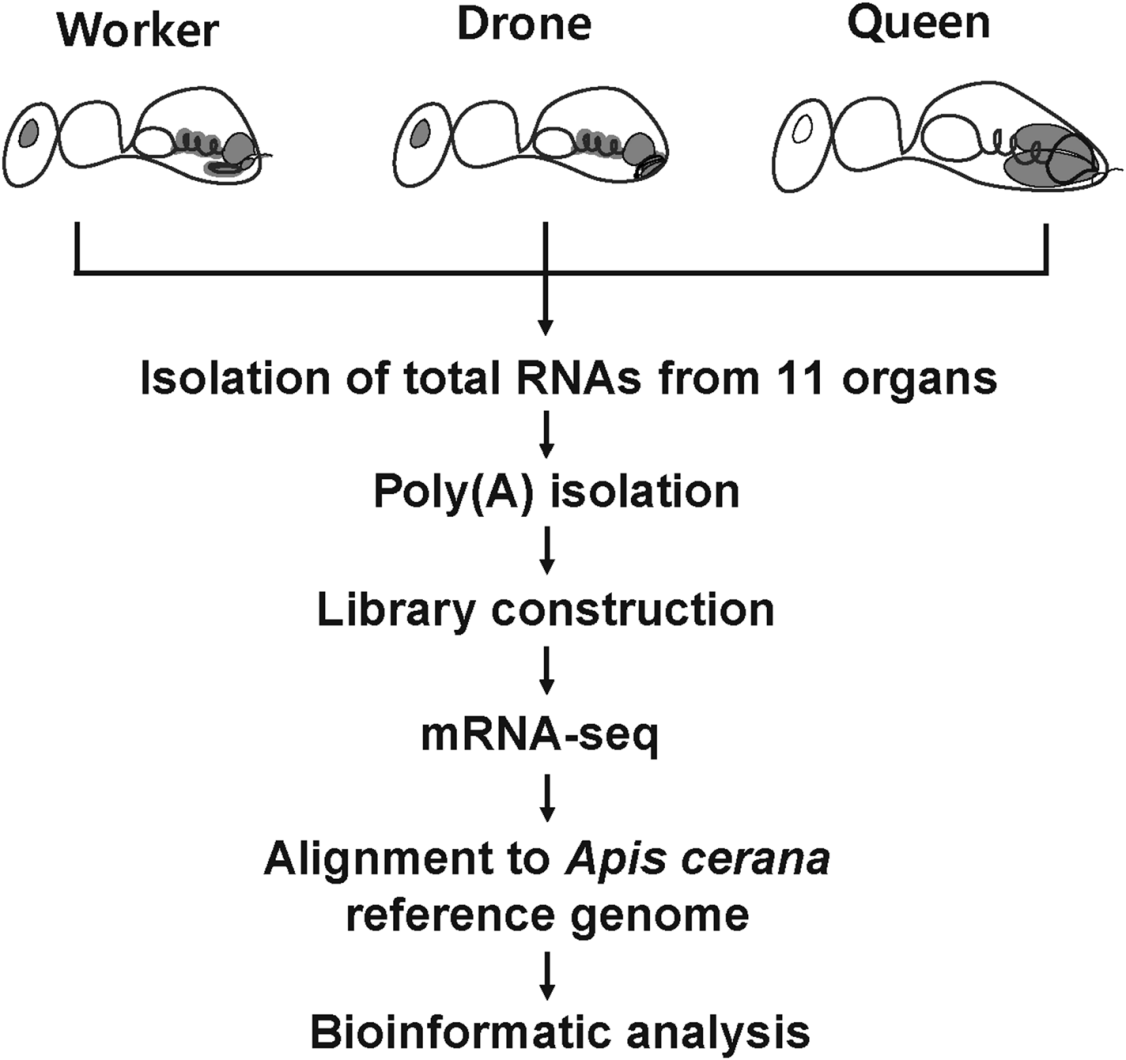
Schematic overview of the experimental design and analysis pipeline. Organs collected directly from adult honeybees for mRNA-seq: brain, ventriculus, rectum from workers and drones, venom gland from worker, and reproductive organs (testis, mucus gland, and ovaries) from three castes. The collected organs shown in gray.

High-throughput sequencing was performed on several organs of *A. cerana*: worker brain (WB), worker ventriculus (WV), worker rectum (WR), worker ovary (WO), worker venom gland (WVG), drone brain (DB), drone ventriculus (DV), drone rectum (DR), drone testis (DT), drone mucus gland (DMG), and queen ovary (QO). High-throughput sequencing was performed using the Illumina HiSeq 2500 platform (Fig. 1). The sequencing results showed that the two biological replicates of each sample have an average of 14 million reads. After removing the adaptor and low-quality tags, approximately 92% clean reads were obtained from each library (Supplementary Table S1).

A comparison of the genomes of *A. cerana* with two other species of insects, i.e., *Drosophila melanogaster* and *A. mellifera* in the NCBI v2.0 database (ACSNU-2.0), provided the coding domain sequence and the read count for gene expression of *A. cerana* using the Kallisto software. The result showed a total of 10,651 genes. High-throughput sequencing analysis indicated that worker, drone, and queen bees had differentially expressed genes. After removing the hypothetical protein, ribosomal protein family, and genes having less than 10 transcripts per million (TPM) reads, a total of 4,096 differentially expressed genes were found among all three castes. Raw and processed data are publicly available in the NCBI/GEO database: (http://www.ncbi.nlm.nih.gov/geo/) under accession number GSE164333.

### Common transcript patterns between worker-drone, worker-queen, and drone-queen

**Figure 2:**
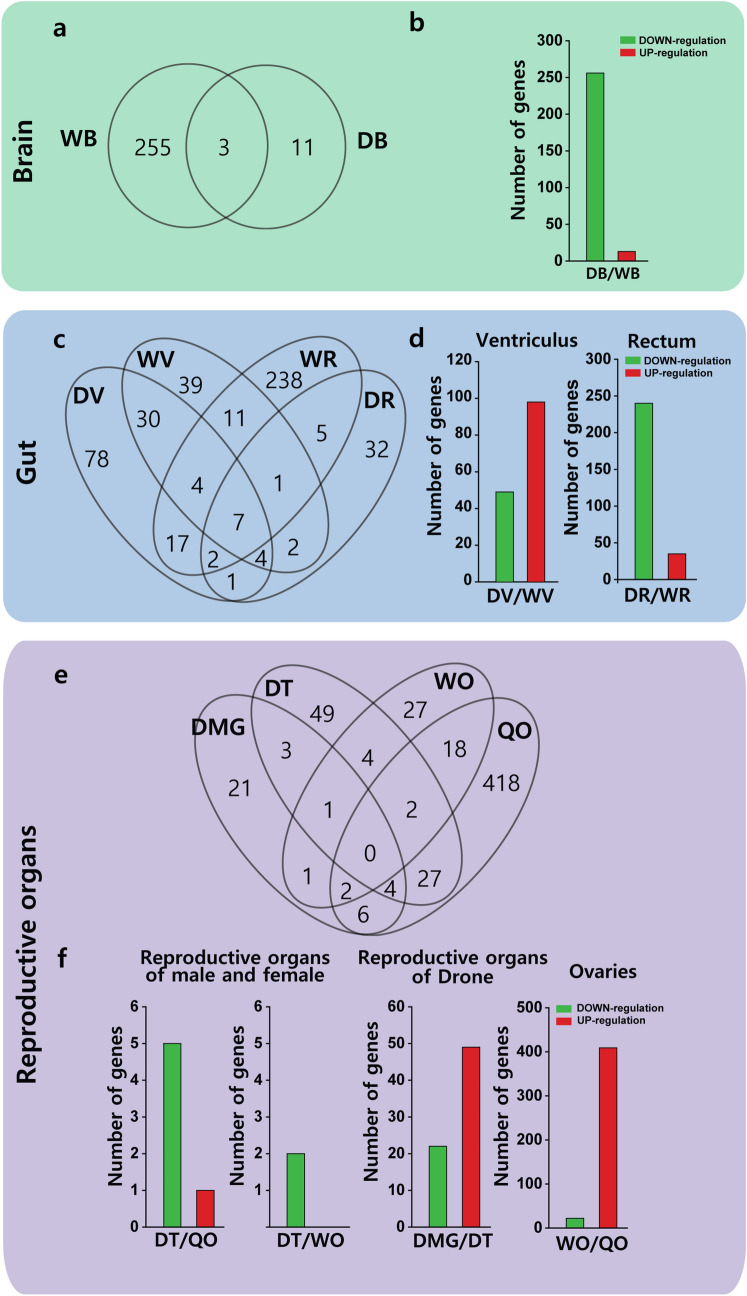
Venn diagram of gene numbers among organs. (**a**) Venn diagram of brain genes between workers and drones. (**b**) Number of down-regulated and up-regulated brain genes between workers and drones. (**c**) Venn diagram of gut genes between worker and drone organs. (**d**) Number of down-regulated and up-regulated gut genes between workers and drones. (**e**) Venn diagram of genes between female and male reproductive organs. (**f**) Number of down-regulated and up-regulated genes between female and male reproductive organs. *WB* worker brain, *WV* worker ventriculus, *WR* worker rectum, *WO* worker ovary, *WVG* worker venom gland, *DB* drone brain, *DV* drone ventriculus (midgut), *DR* drone rectum, *DMG* drone mucus gland, *DT* drone testis, *QO* queen ovary.

One of the major goals of our study was to obtain high-quality transcriptome data that could be used to predict global changes in the gene expressions of honeybees under natural conditions. Transcriptome profiling showed 269, 196, and 324 genes differentially expressed in the brain, ventriculus, and rectum, respectively, of workers and drones. Venn diagram data showed a total of 269 brain-associated genes in WB and DB, among which 255 genes were only expressed in WB, 11 genes were only expressed in DB, and only three genes were expressed commonly in both WB and DB (Fig. 2a,b). Among the 269 genes in brain, 14 genes were correlated with caste differentiation such as hormone, visual sense, and neuronal signal peptides. Among these genes, ten genes, including odorant receptor (OR) genes were up-regulated in WB. The differential expression of transcripts in the brain indicates that they affect honeybee behaviors^29,43,44^. These results also suggest that workers can sense a wider range of odors types.

Differences in the diets of each caste reportedly affect gut enzymes^45^. We found 39, 78, 238, and 32 genes specifically expressed in WV, DV, WR, and DR, respectively. Among these genes, 30 genes were exclusively expressed in ventriculus, whereas, only five genes were expressed in the rectums of both worker and drone (Fig. 2c). In ventriculus, more transcripts were overexpressed in DV compared to WV (Fig. 2d). In the rectum, however, more transcripts were overexpressed in WR compared to DR (Fig. 2d). The ventriculus and rectum, which are typical digestive organs of honeybees, showed substantial differences in the gene expression profile transcripts, despite being anatomically connected.

The reproductive organs of drones (haploid), workers (diploid), and queen (diploid) were compared. Three commonly expressed genes were found in both DT and DMG, and 18 commonly expressed genes in both WO and QO. The numbers of specific transcripts expressed in DT, DMG, WO, and QO were found in 49, 21, 27, and 418 genes, respectively (Fig. 2e). Transcript profiling between DMG and DT revealed that more genes were overexpressed in DT compared to DMG (Fig. 2f). In the comparison between QO and WO as the representative female reproductive organ, most transcripts were overexpressed in QO than in WO (Fig. 2f).

### Differentially expressed genes in the brain, gut, and reproductive organs

**Figure 3:**
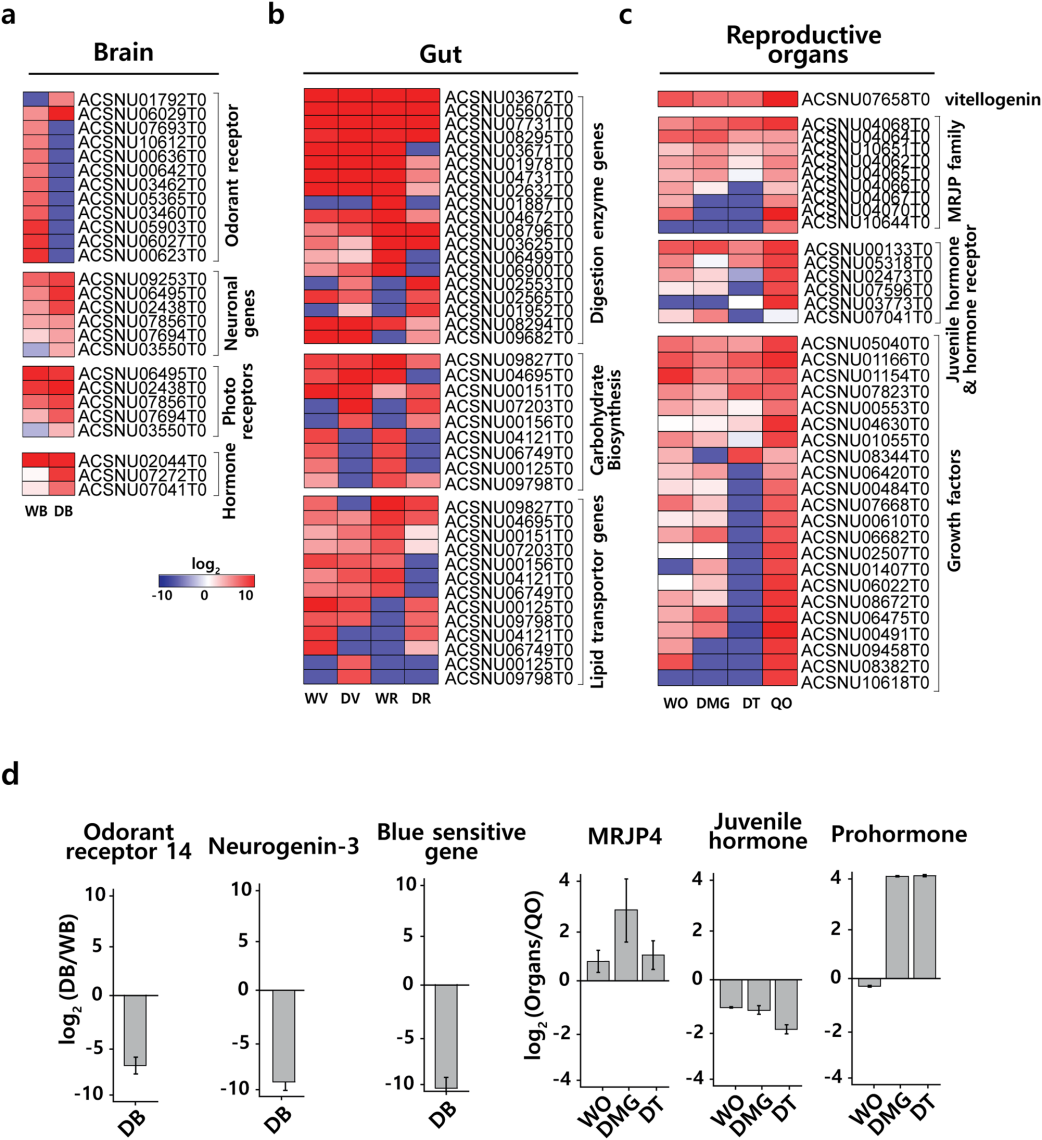
Heat map of transcripts. (**a**) Expression of 26 selected genes among worker and drone brains. 12 genes were related to the odorant receptor family. Six genes were neuronal genes. Five genes were related to vision. Three genes were related to hormone synthesis or hormone peptides. (**b**) Expression of 41 selected genes among worker and drone guts. 19 genes were related the digestive enzyme. Nine genes were related to carbohydrate synthesis. 13 genes were related to lipids. (**c**) Expression of 37 selected genes among worker, drone and queen of reproductive organs. Nine of the major royal jelly protein (MRJP) family. Six genes were related to the juvenile hormone or hormone receptor. 22 genes were related to the growth factor. Each selected gene presented a log_2_ fold change. (**d**) Relative expression of brain related genes and reproductive organ related genes exhibiting a significant change. See Supplementary Table S5 for primers. *WB* worker brain, *WV* worker ventriculus, *WR* worker rectum, *WO* worker ovary, *WVG* worker venom gland, *DB* drone brain, *DV* drone ventriculus (midgut), *DR* drone rectum, *DMG* drone mucus gland, *DT* drone testis, *QO* queen ovary.

To understand the honeybee’s different caste-specific behaviors several categories of genes related to social communication were investigated. The expression patterns of genes were compared among the worker, drone, and queen. Genes from the brain were classified genes into four categories; OR, neuronal genes, photoreceptors, and hormones (Fig. 3a and Supplementary Table S2). A comparison of the OR genes between WB and DB showed remarkably high expression profiles in WB. However, it remains to be determined how each OR affects the honeybee’s behavior. In the other two groups (neuronal genes group and photoreceptor group), the genes related with each group were expressed more in DB than in WB. Of the hormone group, prohormone-1 (ACSNU02044T0) was the only hormone gene that was overexpressed in WB.

We classified gut genes into three categories digestive enzyme genes, carbohydrate biosynthesis genes, and lipid transport genes. In a previous study of the honey bee protein atlas^19^, major digestive enzymes were highly expressed in workers among castes. Comparing these to our data, we found that transcripts of the digestive enzyme were expressed mostly in the rectum of workers (Fig. 3b). The differential profiles of digestive enzyme genes between the worker and drone suggest that there are differences between the diet composition of the two castes.

To understand the difference between haploid and diploid, we collected and examined the gene expressions of four different reproductive organs of worker, drone and queen (Fig. 3c). Vitellogenin is a precursor protein of egg yolk that is used as a biomarker in female^46–48^. In reproductive organs, we found that vitellogenin was expressed in all four reproductive organs. It was most highly expressed in QO, followed by WO, DMG, and DT (Fig. 3c). In a previous data, vitellogenin was first detected in the queen at the mid-late pupal stage, in the worker at the late pupal stage, and in the drone at the adult emergence stage^49^. We classified the genes of reproductive organs into three categories of major royal jelly protein (MRJP) family, hormone-related genes, and growth factors (Fig. 3c and Supplementary Table S2), which are key regulatory factors during early development. When comparing QO with WO, DMG, and DT, transcripts were mostly expressed in QO (Fig. 3c). To validate the result of RNA-seq data, we randomly selected six genes from the brain and reproductive organs and determined their transcription levels using qRT-PCR. The expression patterns of the six genes based on qRT-PCR showed the same patterns (Fig. 3d), which validated the reliability and reproducibility of the RNA-seq data.

### Distribution of the immune system across organ types under natural conditions

**Figure 4:**
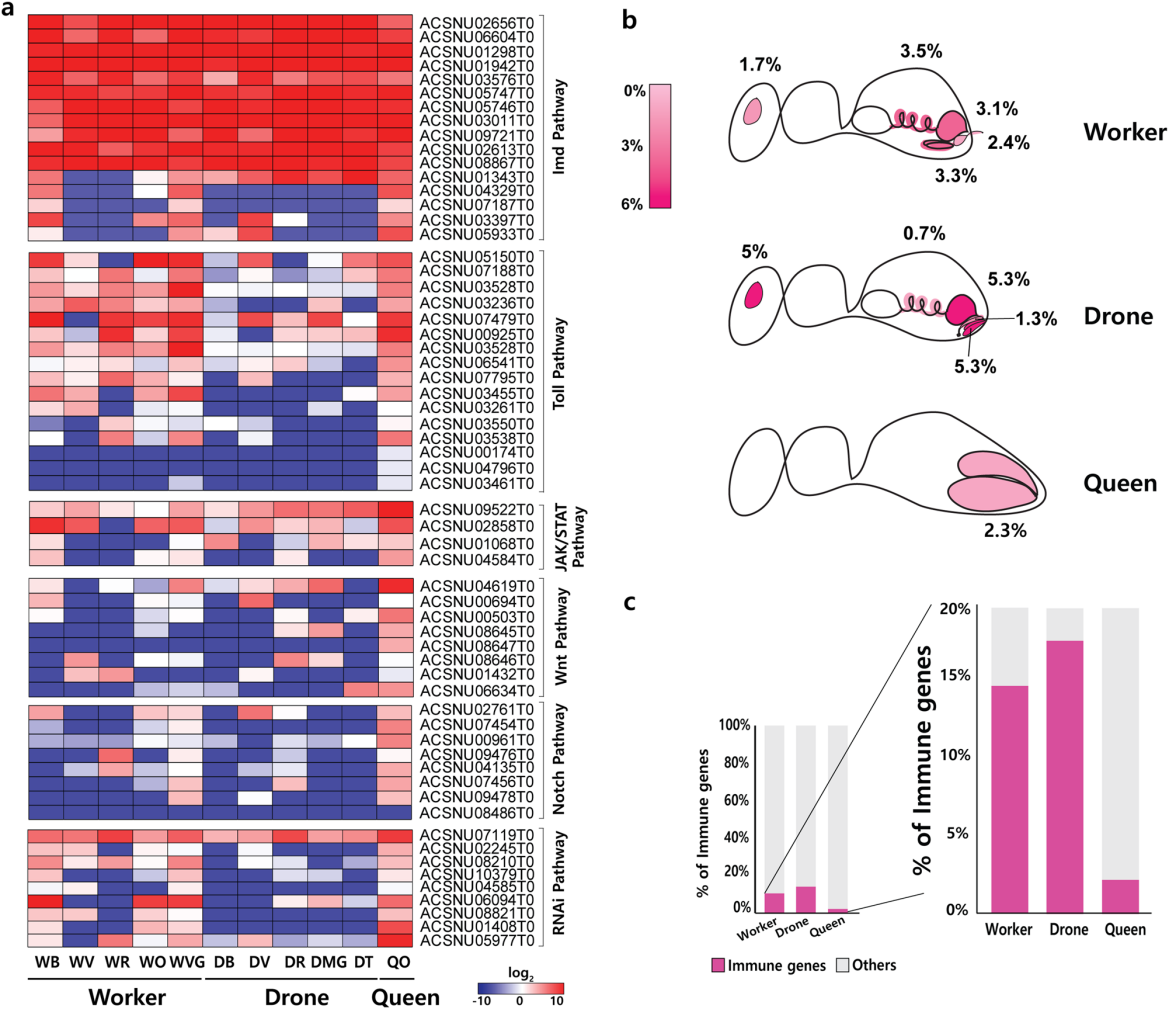
Differences in immune system transcripts across castes. (**a**) Heat map of individual immune genes. (**b**) Distribution of immune-related transcripts in the organs of each caste shown on a pink-scale. Expression ratio showing the proportion of immune-related transcripts expressed among organs in each caste. (**c**) Immune genes as a fraction of all transcripts across castes. The collected organs are shown. *WB* worker brain, *WV* worker ventriculus, *WR* worker rectum, *WO* worker ovary, *WVG* worker venom gland, *DB* drone brain, *DV* drone ventriculus (midgut), *DR* drone rectum, *DMG* drone mucus gland, *DT* drone testis, *QO* queen ovary.

To understand the innate immune system of *A. cerana*, we analyzed the differences in the expression patterns of immune-related genes between castes and displayed them in a heat map (Fig. 4a and Supplementary Table S3). We compared 61 immune-related gene expressions in several specific castes. We found that six immune-response peptides (apidaecin, apidermin, hymenoptaecin, hexamerin, defensin, and T-cell immunomodulatory protein) were highly expressed in all the examined organs. It is well-known that apidaecin, apidermin, hymenoptaecin, hexamerin, and defensin are typical AMPs that correspond to the Toll pathway or Imd pathway. Genes related to the Wnt-signal pathway and RNAi pathway showed very low expression patterns of immune-response genes in the reproductive organs of drones and in the guts of workers and drones (Fig. 4a).

Among the entire gene expressions of different organs from each caste, we investigated the expression ratio of immune-response genes relative to whole transcripts. In comparing the gene expressions, which are displayed in Fig. 4a, the rate of the expression of immune-related genes was different in each organ, which implies that these genes were mainly abundant in the intestinal organs (Fig. 4b). In comparing the transcriptome profiles of whole organs, queen exhibited weakest innate immune-related genes expressed in natural conditions (Fig. 4c).

### Expression of detoxification machinery

**Figure 5:**
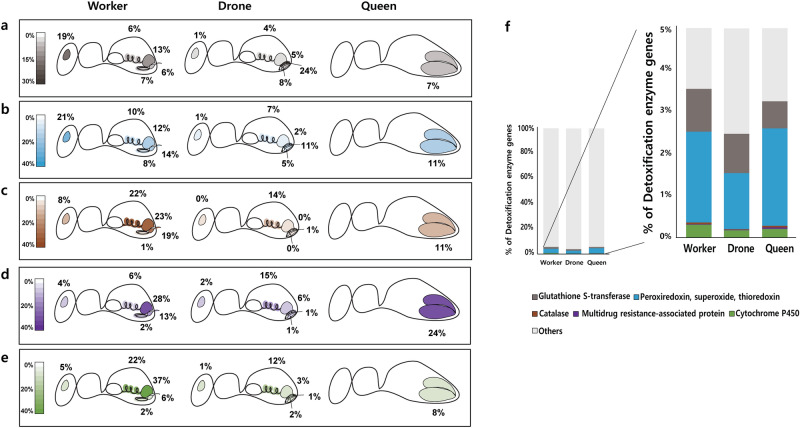
Expression differences in detoxification machineries among castes. Caste and organ distribution of detoxification machineries genes (**a**) Glutathione-S-transferases (GST) transcripts (shown in gray). (**b**) Peroxiredoxin, superoxide, superoxide and thioredoxin transcripts (shown in blue). (**c**) Catalase (shown in brown). (**d**) Multidrug resistance-associated protein (shown in purple). (**e**) Cytochrome P450 (shown in green). Expression ratio showed the proportion of detoxification-related transcripts expressed among organs in each caste (a-e). The collected organs are shown. Modified illustration of the honeybees was adapted with permission from Professor Leonard J. Foster, the corresponding author of Genome Research (2013), 23, 1951–1960. (**f**) The percent of all quantified detoxification machineries in each caste.

We investigated the expression profile of detoxification machinery in unstressed honeybees. In the wild, forager bees collect pollen and transport them to the colony and nurse bees serve nectar as their nutrient resource to feed the brood. Therefore, it is important to detoxify toxic substances such as pesticides that can contaminate the dietary source of the colony. As the detoxification mechanism is assumed to differ among worker, drone, and queen, we compared the detoxification-related expression patterns of five gene families between different castes under natural conditions. First, there were 12 genes in the glutathione-S-transferase family. In order of high expression ratios were 24% in DT, 19% in WB, and 13% in WR (Fig. 5a). Second, there were 39 genes corresponding to oxidation-related biomolecules (peroxiredoxin, superoxide, superoxide and thioredoxin transcripts). We observed the highest expression ratio in WB was 21% (Fig. 5b). Third, there were two genes in catalase. For which the expression ratios were 23% and 22% in WR and WV, respectively (Fig. 5c). Fourth, there were seven genes in the multidrug resistance-associated protein family. In order of high expression, the ratios were 28% and 24% in WR and QO, respectively (Fig. 5d). Fifth, there were 25 genes in the cytochrome P450 transcripts family. The high expression ratio was 37% in WR, then 22% in WV (Fig. 5e). Genes related to detoxification generally showed high expression rates in WV and WR, which may be closely related to the behavior of the worker acting as nurse, server, and pollinator. Compared to the entire transcripts of each organ, genes encoding peroxiredoxin, superoxide, and thioredoxin were expressed ubiquitously with no organ or caste-specific bias (Fig. 5f), which is a similar pattern to the previous proteome report on *A. mellifera*^19^.

### Differences in the haplodiploid sex-determination system between female ovaries

**Figure 6:**
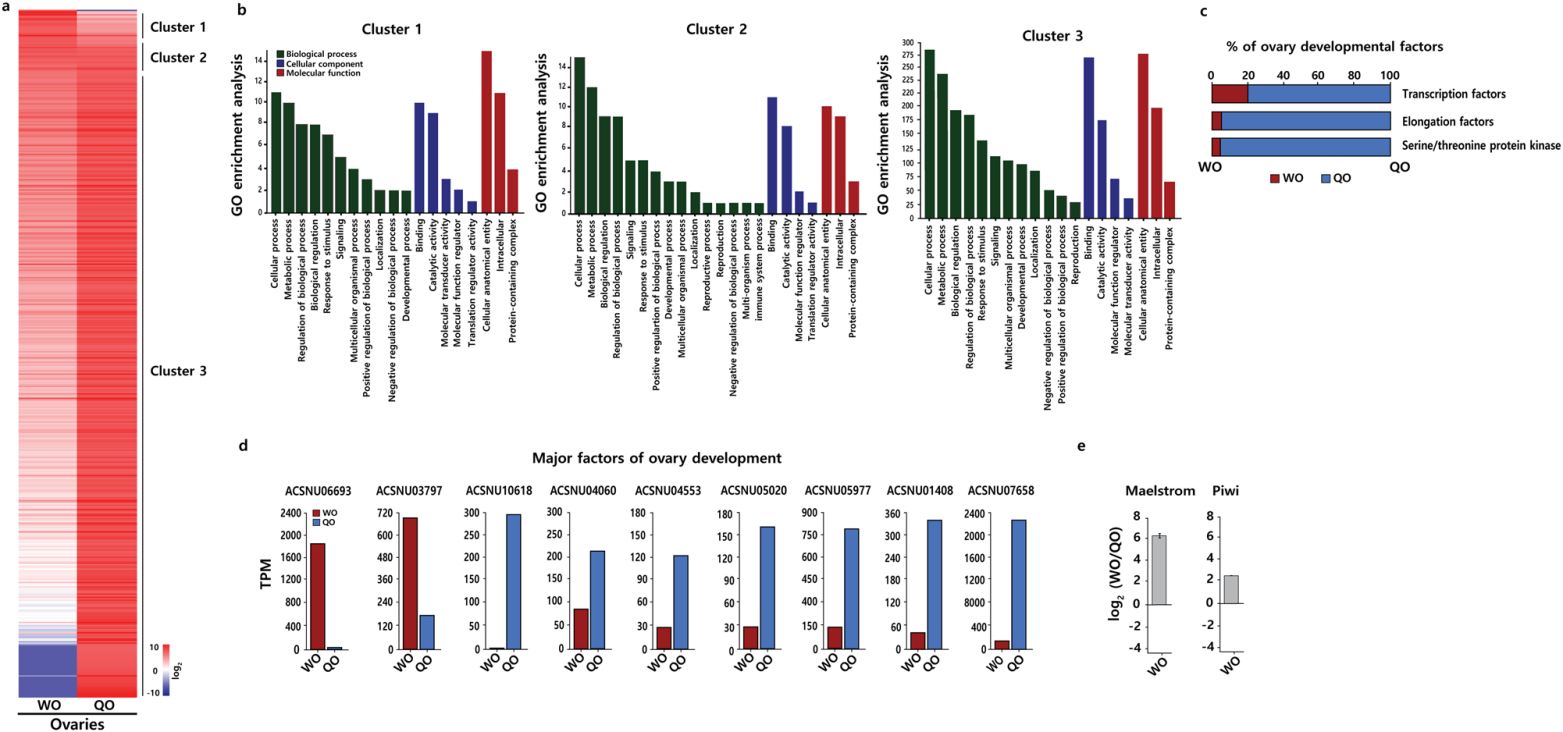
Differences in female ovary transcripts. (**a**) Heat map of WO and QO. Clustering transcripts of ovary (**b**) GO enrichment graph of clusters: biological process (shown in green bars), cellular component (shown in blue bars), and molecular function (shown in red bars). (**c**) The percent expression of ovary development factors in WO (shown in red bars) and QO (shown in blue bars). (**d**) Select nine genes related to the major factors of ovary development. (**e**) Relative expressions of ovary development related genes exhibiting significant change. *WO* worker ovary, *QO* queen ovary.

To understand haplodiploid development more comprehensively, we compared transcriptome data between fertile QO and unfertile WO. We found a total of 463 genes and displayed them on a heat map (Fig. 6a). These genes were grouped into three clusters. We classified the genes up-regulated in WO as Cluster 1, genes with no difference in expression between QO and WO as Cluster 2, and genes up-regulated in QO as Cluster 3 (Supplementary Table S4). To obtain information about the biological processes, cellular components, and molecular function of each gene cluster, we analyzed the gene ontology (GO) in three clusters using BLAST2GO program. GO analysis indicated that genes in Cluster 1 were related to the development process, genes in Cluster 2 were related to the development process and immune system process, and genes in Cluster 3 were related to the development process and reproduction (Fig. 6b). Our results showed that both QO and WO were related to the development process, but only QO was related to reproduction process.

In molecular biology, transcription factors and elongation factors are key regulators in the transcription of genetic processes^50^. Serine/threonine protein kinase plays a significant role in post-translational modification^51^. The proteins that are encoded by these genes are essential for the differentiation and proliferation of germ cells as ovary development factors. In the comparison between QO and WO, QO exhibited extremely large proportions of these factors (Fig. 6c). The transcription levels of nine selected genes encoding for major factors of ovary development can be seen in Fig. 6d. Two of the major factors were up-regulated in WO but others were up-regulated in QO (Fig. 6d). To determine the fold changes in the expression levels of two selected genes of *maelstrom* and *piwi* between QO and WO, the results from qRT-PCR analysis matched well with the RNA-seq results (Fig. 6e), which validated the reliability and reproducibility of RNA-seq data.

### Expression patterns of venom encoding genes in organs

**Figure 7:**
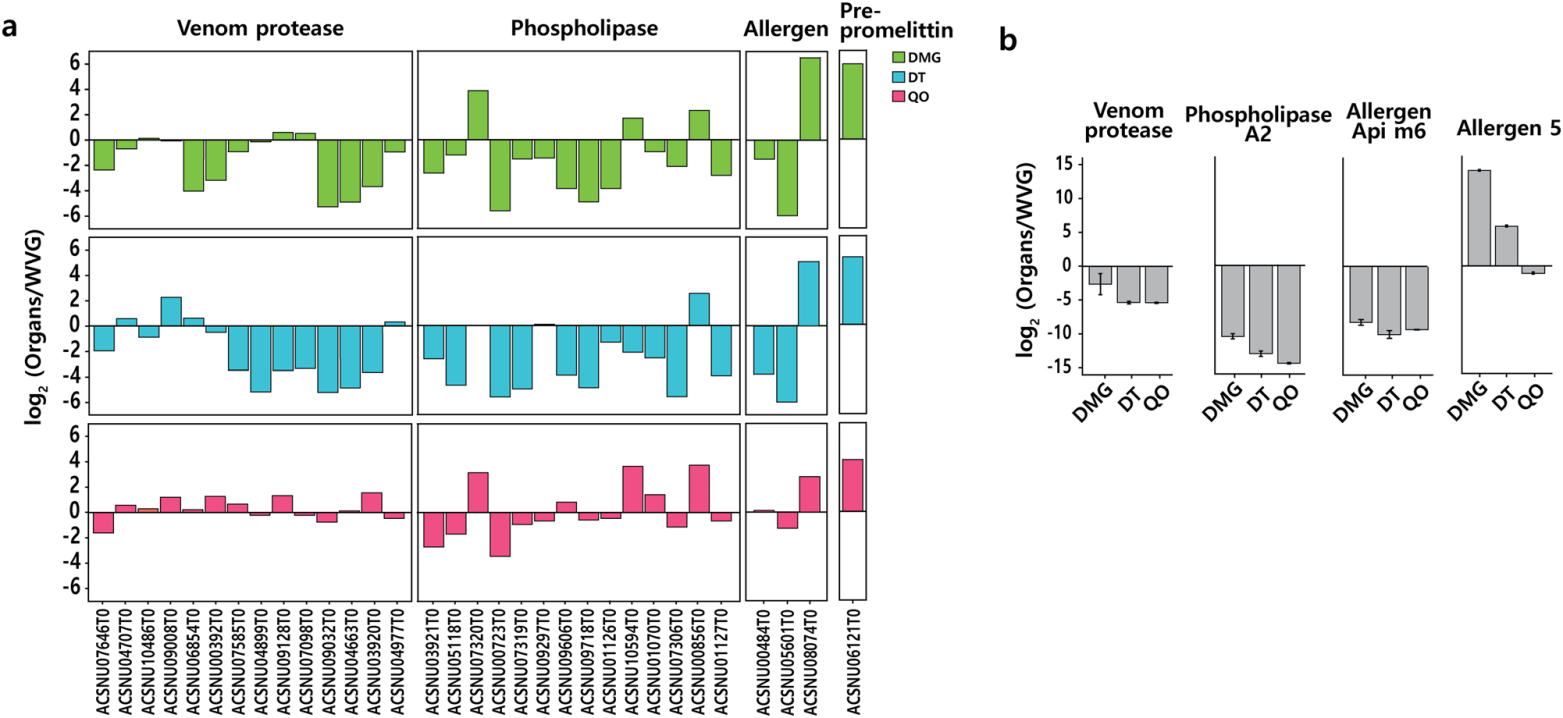
Expressions differences of venom-specific genes among reproductive organs. (**a**) Values above zero indicated greater relative transcript abundance in reproductive organs compared that of to the venom gland. Genes were differentially expressed at a *p*-value < 0.05. (**b**) Relative expressions of venom-specific genes exhibiting significant change. *DMG* drone mucus gland, *DT* drone testis, *QO* queen ovary.

There were 105 genes related to venom processes. Initially, high expression patterns were expected for the 105 venom-related genes in WVG, but this was not the case. The expressions of the 105 venom encoding genes in the reproductive organs of the drone and queen were compared with those of the WVG. As expected, genes related to venom protease and phospholipase were generally highly expressed in WVG. However, some venom genes, including phospholipase, prepromelittin gene (which is required to produce melittin), and the venom allergen 5-like (ACSNU08074T0), which is a toxic peptide, showed higher expressions in the drone and queen reproductive organs (Fig. 7a). The mRNA-seq results using qRT-PCR revealed the same patterns for four randomly selected genes (Fig. 7b). These results validate the reliability and reproducibility of RNA-seq data.

## Discussion

Here, we presented high-quality transcriptome profiles of organs from different caste members of the *A. cerana*. In the data set, our results showed differences in transcripts between female and male honeybees in natural conditions. Furthermore, our transcriptome analysis of *A. cerana*, provides an important resource for future molecular studies of Asian honeybees, particularly to elucidate the mechanisms of the innate immune system, social behavior, genetic difference between females (worker and queen) and male (drone), and the potential function of haplodiploid development.

AMPs are effective against pathogens that are frequently encountered in honeybees hive^33^. Our data demonstrate that some AMPs were highly expressed in DR, and DMG. The results indicate that AMPs and detoxification-related genes were expressed differently among castes. The expression of AMPs-related genes was mainly expressed, while. detoxification genes were low expression in drones. However, both of the AMPs-related genes and detoxification genes were highly expressed in workers. Therefore, we presume that workers possess immunity against bacteria that are introduced from the outside while foraging outside as a pollinator or as nursing brood to feed pollen. Previous studies showed the workers infected by bacteria had a strong immune response^38^.

It has been reported that the nutrition and dietary habits of bees affect their physiology^52^. Therefore, in the present study, we compared gene expression patterns between worker guts and drone guts. Detoxification mechanisms in honeybee guts, which help bees develop tolerate to a variety of potentially toxic secondary metabolites and pesticides that they encounter in floral nectars and pollen, remain largely unknown^53^. The latest study on the protein atlas of honey bee organs^19^ represented the robust detoxification machinery of worker organs. However, our expression data shows that the detoxification-related transcriptome expressed more in the drone, than in the worker. In this study, we provide information on these observed transcriptional changes, which are not reflected in data that was obtained on the protein level^19^.

The honeybee has a unique sex-determination system that is also known as haplodiploidy. Unfertilized eggs are haploid, whereas fertilized eggs are diploid^54^. Haploid eggs develop into males, whereas diploid eggs develop into females. Vitellogenin is a precursor protein of egg yolk that is used as a biomarker in female^46–48^ and is also expressed in the fat bodies of workers and queens^55^. In a previous study, the vitellogenin protein played an important role in ovary development, immunity, stress response, and sex-determination^46–48,56^. In our results, vitellogenin was expressed in both QO and WO, but transcripts related with ovary development were highly expressed in QO.

Interestingly, the *maelstrom* transcript and *piwi* protein transcript were found in both QO and WO. Mael and Piwi proteins are required for transcriptional silencing, which is induced by the piRNA pathway. Our results showed that different expressions of *mael* and *piwi* were observed in QO and WO. The Mael protein repressed canonical RNA polymerase II transcription and inhibits germline transposon transcription^57,58^. Based on previous papers and our recent findings, we propose that *mael* and *piwi* play an important role in the development of QO and WO.

Among all organs, venom-producing organs give honeybees the ability to protect their colonies from enemies by using venom as a weapon^59^. The WVG produces several components of honeybee venom that cause allergic responses in humans^60^ and other vertebrates. Honeybee venom contains the major allergens of Api m 1 (phospholipase A2), Api m 2 (hyaluronidase), Api m 3 (acid phosphatase), Api m 4 (melittin), and Api m 7 (CUB serine protease). Melittin, the main constituent of honeybee venom, is one of the major proteins that composes venom toxicity^61^ and is derived from promelittin^62,63^. However, it was not unknown how prepromelittin is converted into promelittin. In our results, prepromelittin was proven to be transcribed in the reproductive organs of drones and queen. Therefore, it is speculated that cleavage occurs with melittin after the gene is transferred and that biosynthesis of melittin from prepromelittin might occur after the prepromelittin gene is transferred from the parent to the worker bee. Our data do not clearly demonstrate this hypothesis, but we provide a clue that indicates the synthesis process of the melittin, which guides future research.

In this study, we investigated the transcriptome profiles in several organs from different castes to determine unique traits. The results revealed that immune-related genes and detoxification mechanisms were generally highly expressed in workers in natural conditions. Thus, we provide a foundation for understanding the physiology, morphology, and caste formation of *A. cerana* at the molecular level. We hope that this study will stimulate future research on the Asian honeybee, which is less understood compared to Western honeybees.

## Methods

### Sample collection

Samples from each developmental stage of *A. cerana* were collected from colonies kept in Cheonan, South Korea. To obtain for the samples, one colony of a mated egg-laying queen was used. Bees were collected from the colony during swarm season, and placed in a 1.5 ml microfuge tube. Prior to dissection, the bees were exposed to CO_2_ in order to aid handling. Each organ was dissected using forceps and microdissection scissors. All dissected organs were rinsed with PBS solution^64^. Each dissected organ sample was flash-frozen in liquid nitrogen and then stored at − 80 °C. Organs were obtained from three castes of the *A. cerana* i.e., worker, drone, queen. Worker organs included worker brain (WB), worker ventriculus (WV), worker rectum (WR), and worker venom gland (WVG). Drone organs include drone brain (DB), drone ventriculus (DV), drone rectum (DR), drone testis (DT), and drone mucus gland (DMG). Queen organ includes queen ovary (QO).

### Preparation of the mRNA-sequencing library

Total RNA was extracted from the organ samples using TRIzol reagent (Invitrogen). An equal amount of 5 μg total RNA from each sample was used to construct the mRNA library according to manufacturer instructions (Lexogen, Austria). The libraries were sequenced at a high-throughput sequencing facility (Macrogen, South Korea) on an Illumina HiSeq 2500 platform. The sequencing data were obtained with two biological replicates of each organ.

### Computational analysis

The raw Illumina sequence reads were filtered to remove low quality sequences by using fastp^65^ prior to bioinformatic analysis. The filtered Illumina short reads were then mapped to the *A. cerana* reference genome (ACSNU-2.0)^66^ using Bowtie2 software^67^. The mapping results of each sample to the reference mRNA sequences obtained by Bowtie2 were then quantified by Kallisto software^68^. The gene expression profiles of each sample obtained by Kallisto were calculated as TPM value to compare the gene expression levels between samples.

### Validation of differentially expressed genes by quantitative real-time PCR

The relative mRNA levels were quantified according to the manufacturer’s instruction using an AccuPower 2X GreenStar qPCR Master Mix (Bioneer, South Korea). Results were normalized to actin mRNA. Gene expression levels were calculated using the comparative Ct method. All experiments were carried out at least three times for each of three biological replicates. The gene-specific primers used for qRT-PCR are listed in Supplementary Table S5.

## Supplementary Information

Supplementary Information.

## Data Availability

The datasets generated for this study can be found in the NCBI SRA repository, https://www.ncbi.nlm.nih.gov/sra/, with the GEO Accession No.: GSE164333.

## References

[1] Garibaldi LA (2013). Wild pollinators enhance fruit set of crops regardless of honey bee abundance. Science.

[2] Southwick EE, Southwick L (1992). Estimating the economic value of honey bees (Hymenoptera: Apidae) as agricultural pollinators in the United States. J. Econ. Entomol..

[3] Magrach A, González-Varo JP, Boiffier M, Vilà M, Bartomeus I (2017). Honeybee spillover reshuffles pollinator diets and affects plant reproductive success. Nat. Ecol. Evol..

[4] Corbet SA, Williams IH, Osborne JL (1991). Bees and the pollination of crops and wild flowers in the European Community. Bee World.

[5] Herrera CM, de Vega C, Canto A, Pozo MI (2009). Yeasts in floral nectar: A quantitative survey. Ann. Bot..

[6] Hossen MS (2017). Beneficial roles of honey polyphenols against some human degenerative diseases: A review. Pharmacol. Rep..

[7] Cheng PC, Wong G (1996). Honey bee propolis: Prospects in medicine. Bee World.

[8] Lerrer B, Zinger-Yosovich KD, Avrahami B, Gilboa-Garber N (2007). Honey and royal jelly, like human milk, abrogate lectin-dependent infection-preceding *Pseudomonas aeruginosa* adhesion. ISME J..

[9] Alvarez-Suarez JM, Tulipani S, Romandini S, Bertoli E, Battino M (2010). Contribution of honey in nutrition and human health: A review. Mediterr. J. Nutr. Metab..

[10] Wu J (2011). Caffeic acid phenethyl ester (CAPE), derived from a honeybee product propolis, exhibits a diversity of anti-tumor effects in pre-clinical models of human breast cancer. Cancer Lett..

[11] Viuda-Martos M, Ruiz-Navajas Y, Fernández-López J, Pérez-Álvarez J (2008). Functional properties of honey, propolis, and royal jelly. J. Food Sci..

[12] Tsuruda JM, Amdam GV, Page RE (2008). Sensory response system of social behavior tied to female reproductive traits. PLoS ONE.

[13] Greenberg JK (2012). Behavioral plasticity in honey bees is associated with differences in brain microRNA transcriptome. Genes Brain Behav.

[14] Tan K (2015). A neonicotinoid impairs olfactory learning in Asian honey bees (Apis cerana) exposed as larvae or as adults. Sci Rep.

[15] Ambros V (2003). A uniform system for microRNA annotation. RNA.

[16] Ament SA (2012). The transcription factor ultraspiracle influences honey bee social behavior and behavior-related gene expression. PLoS Genet.

[17] Okosun OO, Pirk CW, Crewe RM, Yusuf AA (2017). Glandular sources of pheromones used to control host workers (*Apis mellifera scutellata*) by socially parasitic workers of *Apis mellifera* capensis. J. Insect Physiol..

[18] Keeling CI, Slessor KN, Higo HA, Winston ML (2003). New components of the honey bee (*Apis mellifera* L.) queen retinue pheromone. Proc. Natl. Acad. Sci..

[19] Chan QW (2013). Honey bee protein atlas at organ-level resolution. Genome Res..

[20] Ashby R, Forêt S, Searle I, Maleszka R (2016). MicroRNAs in honey bee caste determination. Sci. Rep..

[21] Collins DH (2017). MicroRNAs associated with caste determination and differentiation in a primitively eusocial insect. Sci. Rep..

[22] Pires CV, Freitas FCDP, Cristino AS, Dearden PK, Simões ZLP (2016). Transcriptome analysis of honeybee (*Apis Mellifera*) haploid and diploid embryos reveals early zygotic transcription during cleavage. PLoS ONE.

[23] Shi YY (2011). Diet and cell size both affect queen-worker differentiation through DNA methylation in honey bees (*Apis mellifera*, Apidae). PLoS ONE.

[24] Diao Q (2018). Genomic and transcriptomic analysis of the Asian honeybee *Apis cerana* provides novel insights into honeybee biology. Sci. Rep..

[25] Forêt S, Maleszka R (2006). Function and evolution of a gene family encoding odorant binding-like proteins in a social insect, the honey bee (*Apis mellifera*). Genome Res..

[26] Smith BH (1991). The olfactory memory of the honeybee Apis mellifera: I. Odorant modulation of short-and intermediate-term memory after single-trial conditioning. J. Exp. Biol..

[27] Calvello M (2005). Expression of odorant-binding proteins and chemosensory proteins in some Hymenoptera. Insect Biochem. Mol. Biol..

[28] Grozinger CM, Sharabash NM, Whitfield CW, Robinson GE (2003). Pheromone-mediated gene expression in the honey bee brain. Proc. Natl. Acad. Sci..

[29] Sasaki K, Akasaka S, Mezawa R, Shimada K, Maekawa K (2012). Regulation of the brain dopaminergic system by juvenile hormone in honey bee males (*Apis mellifera* L.). Insect Mol. Biol..

[30] Kamakura M (2011). Royalactin induces queen differentiation in honeybees. Nature.

[31] Corona M (2007). Vitellogenin, juvenile hormone, insulin signaling, and queen honey bee longevity. Proc. Natl. Acad. Sci..

[32] DeGrandi-Hoffman G, Chen Y (2015). Nutrition, immunity and viral infections in honey bees. Curr. Opin. Insect Sci..

[33] Danihlík J, Aronstein K, Petřivalský M (2015). Antimicrobial peptides: A key component of honey bee innate immunity: Physiology, biochemistry, and chemical ecology. J. Apic. Res..

[34] Kim WJ (2017). Differential gene expressions of innate immune related genes of the Asian honeybee, *Apis cerana*, latently infected with sacbrood virus. J. Asia-Pac. Entomol..

[35] Goode K, Huber Z, Mesce KA, Spivak M (2006). Hygienic behavior of the honey bee (*Apis mellifera*) is independent of sucrose responsiveness and foraging ontogeny. Horm. Behav..

[36] Harwood G, Salmela H, Freitak D, Amdam G (2021). Social immunity in honey bees: Royal jelly as a vehicle in transferring bacterial pathogen fragments between nestmates. J. Exp. Biol..

[37] Raymann K, Moran NA (2018). The role of the gut microbiome in health and disease of adult honey bee workers. Curr. Opin. Insect Sci..

[38] Bull JC (2012). A strong immune response in young adult honeybees masks their increased susceptibility to infection compared to older bees. PLoS Pathog..

[39] Bastin F, Cholé H, Lafon G, Sandoz J-C (2017). Virgin queen attraction toward males in honey bees. Sci. Rep..

[40] Nunes TM (2014). Queen signals in a stingless bee: Suppression of worker ovary activation and spatial distribution of active compounds. Sci. Rep..

[41] Pirk CW, Neumann P, Hepburn R, Moritz RF, Tautz J (2004). Egg viability and worker policing in honey bees. Proc. Natl. Acad. Sci..

[42] Cowan DP, Stahlhut JK (2004). Functionally reproductive diploid and haploid males in an inbreeding hymenopteran with complementary sex determination. Proc. Natl. Acad. Sci..

[43] Han B (2015). Quantitative neuropeptidome analysis reveals neuropeptides are correlated with social behavior regulation of the honeybee workers. J. Proteome Res..

[44] Townson SM (1998). Honeybee blue-and ultraviolet-sensitive opsins: Cloning, heterologous expression in Drosophila, and physiological characterization. J. Neurosci..

[45] Ellegaard KM, Engel P (2019). Genomic diversity landscape of the honey bee gut microbiota. Nat. Commun..

[46] Amdam GV, Simões ZL, Guidugli KR, Norberg K, Omholt SW (2003). Disruption of vitellogenin gene function in adult honeybees by intra-abdominal injection of double-stranded RNA. BMC Biotechnol..

[47] Awde DN, Skandalis A, Richards MH (2020). Vitellogenin expression corresponds with reproductive status and caste in a primitively eusocial bee. J. Insect Physiol..

[48] Guidugli KR (2005). Vitellogenin regulates hormonal dynamics in the worker caste of a eusocial insect. FEBS Lett..

[49] Piulachs M (2003). The vitellogenin of the honey bee, *Apis mellifera*: Structural analysis of the cDNA and expression studies. Insect Biochem. Mol. Biol..

[50] Yang H, Basquin D, Pauli D, Oliver B (2017). Drosophila melanogaster positive transcriptional elongation factors regulate metabolic and sex-biased expression in adults. BMC Genomics.

[51] Préat T (1990). A putative serine/threonine protein kinase encoded by the segment-polarity fused gene of Drosophila. Nature.

[52] Sarma MS, Whitfield CW, Robinson GE (2007). Species differences in brain gene expression profiles associated with adult behavioral maturation in honey bees. BMC Genomics.

[53] Esther E (2015). Detoxification mechanisms of honey bees (*Apis mellifera*) resulting in tolerance of dietary nicotine. Sci. Rep..

[54] Charlesworth B (2003). Sex determination in the honeybee. Cell.

[55] Kocher SD, Richard F-J, Tarpy DR, Grozinger CM (2008). Genomic analysis of post-mating changes in the honey bee queen (Apis mellifera). BMC Genomics.

[56] Zhang W (2017). Molecular cloning, expression and oxidative stress response of the vitellogenin Gene (AccVg) from *Apis cerana cerana*. Apidologie.

[57] Chang TH (2019). Maelstrom represses canonical polymerase II transcription within bi-directional piRNA clusters in *Drosophila melanogaster*. Mol. Cell.

[58] Pek JW, Lim AK, Kai T (2009). Drosophila maelstrom ensures proper germline stem cell lineage differentiation by repressing microRNA-7. Dev. Cell.

[59] Nouvian M, Reinhard J, Giurfa M (2016). The defensive response of the honeybee *Apis mellifera*. J. Exp. Biol..

[60] Sobotka AK, Kochoumian L, Lichtenstein LM (1976). Allergens of honey bee venom. Arch. Biochem. Biophys..

[61] Fletcher JE, Jiang M-S (1993). Possible mechanisms of action of cobra snake venom cardiotoxins and bee venom melittin. Toxicon.

[62] Hoffman DR (2006). Hymenoptera venom allergens. Clin. Rev. Allergy Immunol..

[63] Mollay C, Vilas U, Kreil G (1982). Cleavage of honeybee prepromelittin by an endoprotease from rat liver microsomes: Identification of intact signal peptide. Proc. Natl. Acad. Sci..

[64] Carreck NL (2013). Standard methods for Apis mellifera anatomy and dissection. J. Apic. Res..

[65] Chen S, Zhou Y, Chen Y, Gu J (2018). fastp: An ultra-fast all-in-one FASTQ preprocessor. Bioinformatics.

[66] Park D (2015). Uncovering the novel characteristics of Asian honey bee, *Apis cerana*, by whole genome sequencing. BMC Genomics.

[67] Langmead B, Salzberg SL (2012). Fast gapped-read alignment with Bowtie 2. Nat. Methods.

[68] Bray NL, Pimentel H, Melsted P, Pachter L (2016). Near-optimal probabilistic RNA-seq quantification. Nat. Biotechnol..

