# Supplementary material for: Organ-specific transcriptome analysis reveals differential gene expression in different castes under natural conditions in Apis cerana
Source: Sci Rep. 2021 May 28;11:11267. doi: 10.1038/s41598-021-90635-3 (PMC8163739; doi:10.1038/s41598-021-90635-3)
Supplement: Supplementary file 1 — Supplementary Information. [file 41598_2021_90635_MOESM1_ESM.zip › Supplementary Dataset/Supplementary information front page_ESM.docx]

**SUPPLEMENTARY INFORMATION**

**Organ-specific transcriptome analysis reveals differential gene expression in different castes under natural conditions in *Apis cerana***

Igojo Kang^1^, Woojin Kim^2^, Jae Yun Lim^1^, Yun Lee^3^ and Chanseok Shin^1,4,5*^

^1^Department of Agricultural Biotechnology, Seoul National University, Seoul 08826, Republic of Korea.

^2^Department of Agricultural Biology, Jeonbuk National University, Jeonju 54896, Republic of Korea.

^3^Department of Applied Biology and Chemistry, Seoul National University, Seoul 08826, Republic of Korea.

^4^Research Institute of Agriculture and Life Sciences, Seoul National University, Seoul 08826, Republic of Korea.

^5^Plant Genomics and Breeding Institute, Seoul National University, Seoul 08826, Republic of Korea.

*Corresponding author:

Chanseok Shin, Department of Agricultural Biotechnology, Institute of Agriculture and Life Sciences, and Plant Genomics and Breeding Institute, Seoul National University, Seoul, Republic of Korea. E-mail: [cshin@snu.ac.kr](mailto:cshin@snu.ac.kr)

**Supplementary Tables**

**Supplementary Table S1.** Statistical summary of RNA-seq data.

**Supplementary Table S2**. Sequencing read counts and annotation of *Apis cerana* from brain, gut, and reproductive organs.

**Supplementary Table S3.** Sequencing read counts and innate immune pathways of *Apis cerana* from three caste organs.

**Supplementary Table S4.** Sequencing read counts and annotation of *Apis cerana* from the two kinds of female ovaries.

**Supplementary Table S5.** Primer information for target genes used in this study.
